# *Candida* Susceptibility to Antifungals in Amniotic Fluid: A Preliminary Study

**DOI:** 10.3390/pathogens14100972

**Published:** 2025-09-25

**Authors:** Silvia Gabriela Ionescu, Cristina Daniela Dimitriu, Demetra Gabriela Socolov, Mihaela Grigore, Luminita Smaranda Iancu, Costin Damian, Roxana Gabriela Cobzaru, Carmen Valerica Ripa, Diana Costin, Radu-Florin Popa, Brindusa Copacianu, Ramona Gabriela Ursu

**Affiliations:** 1 Department of Preventive Medicine and Interdisciplinarity (IX)—Microbiology, Grigore T. Popa University of Medicine and Pharmacy Iași, 700115 Iași, Romania; 2 Department of Biochemistry, Grigore T. Popa University of Medicine and Pharmacy Iași, 700115 Iași, Romania; 3 ”Cuza-Vodă” Clinical Hospital of Obstetrics and Gynecology, 700038 Iași, Romania; 4 Department of Gynecology and Obstetrics, Grigore T. Popa University of Medicine and Pharmacy Iași, 700115 Iași, Romania; 5 Department of Vascular Surgery, Grigore T. Popa University of Medicine and Pharmacy Iași, 700115 Iași, Romania; 6 Microbiology Laboratory, Regional Institute of Oncology, 700483 Iasi, Romania

**Keywords:** *Candida* spp., amniotic fluid, antifungal susceptibility: minimum inhibitory concentration, intra-amniotic infection, real-time PCR

## Abstract

(1) Background: Fungal infections of amniotic fluid, especially those caused by *Candida* spp., are rare but clinically important, as they can be correlated with preterm birth and poor neonatal outcomes. The aim of this study was to assess the antifungal susceptibility of *Candida* spp. isolated from amniotic fluid using an MIC (minimum inhibitory concentration)-based assay. (2) Methods: Forty consecutive, exploratory *Candida* isolates were identified from amniotic fluid samples at the “Cuza Vodă” Clinical Hospital of Obstetrics and Gynecology, Iași, and were analyzed successively using Sabouraud agar, the VITEK^®^ 2 Compact system, and real-time PCR (RT-PCR). (3) Results: *C. albicans* was the most abundant species (67.5%), followed by *Pichia kudriavzevii*, *Nakaseomyces glabratus*, *C. parapsilosis*, and *C. dubliniensis*. Fluconazole resistance was observed in two *C. albicans* isolates, emphasizing the clinical importance of routine antifungal susceptibility testing, and all *C. albicans* isolates were resistant to micafungin, while amphotericin B remained effective against all isolates. RT-PCR confirmed the presence of *C. albicans* DNA. (4) Conclusions: The detection of resistant *Candida* strains highlights the importance of conducting assessments at the species level, which could help clinicians to ensure better antifungal stewardship.

## 1. Introduction

The WHO Fungal Priority Pathogens List (FPPL), published in 2022, categorizes 19 fungal species into three priority groups: critical, high, and medium. This classification is based on factors such as antifungal resistance, mortality, global incidence, and the availability of diagnostics and treatment. The critical group includes major threats like *Cryptococcus neoformans*, *Candida auris*, *Aspergillus fumigatus*, and *Candida albicans*; the high-priority group comprises species such as *Nakaseomyces glabratus*, *Candida tropicalis*, and *Histoplasma* spp.; and the medium category includes *Pichia kudriavzevii*, *Coccidioides* spp., and *Pneumocystis jirovecii* [1].

Several case reports have confirmed the presence of *C. albicans* in amniotic fluid. In one case, prolonged high-dose liposomal amphotericin B extended gestation from 25 to 30 weeks despite persistently positive cultures. Drug levels were higher in maternal and cord blood than in amniotic fluid, suggesting effective transplacental passage and fetal protection, and the neonate was delivered healthy, without invasive fungal infection [2]. Another case reported intra-amniotic *C. albicans* infection following cerclage placement for cervical insufficiency, with persistent positive cultures. The patient declined termination and received systemic plus intra-amniotic fluconazole. Although cultures remained positive, the neonate had no fungemia, suggesting that antifungal therapy may lower fetal risk even without full clearance of *Candida* [3]. Advanced sequencing methods have facilitated better understanding of intrauterine and neonatal infections. One study used next-generation sequencing (NGS) on gastric aspirates from neonates with respiratory distress and identified *C. albicans* in one case. Even though it did not meet positivity thresholds, this still suggests that gastric fluid might be useful in spotting vertically transmitted pathogens [4]. Besides *C. albicans*, other species such as *N. glabratus* have been reported in this context. In a case, the organism was found in both amniotic fluid and maternal blood, even though the serum β-D-glucan test was negative, showing how difficult diagnosis can be and how necessary clinical vigilance is [5]. Cross-sectional studies have provided additional evidence regarding the infectious characteristics of amniotic fluid debris observed in preterm labor [6]. Cultures have often revealed microorganisms like *Ureaplasma*, *Mycoplasma*, and *C. albicans*, especially before 33 weeks of pregnancy, supporting the role of intra-amniotic echogenic material as a marker of intrauterine inflammation [6]. In a retrospective study of very-low-birth-weight infants (VLBWIs) with early-onset sepsis, *C. albicans* was found to have caused a small but not insignificant number of infections. Risk factors included amniotic fluid contamination and maternal fever, again linking prenatal fungal presence to poor neonatal outcomes [7]. An in vitro study demonstrated that term amniotic fluid exhibits antifungal activity against various *Candida* species and completely inhibits *N. glabratus*, suggesting that amniotic fluid potentially has a natural protective role [8]. *Candida auris* is an emerging multidrug-resistant pathogen, designated by the WHO as critical-priority due to its nosocomial outbreaks and frequent antifungal resistance, with over 20 outbreaks reported worldwide from 2019 to 2024. Diagnostic tools for the fungus range from MALDI-TOF and WGS to real-time PCR, which can simultaneously detect species and resistance markers such as FKS (echinocandin resistance) and ERG11 (azole resistance), which can be mutated and/or experience a change in expression [9]. Although intrauterine fungal infections are considered rare, *Candida* spp. may substantially affect obstetric and neonatal outcomes, inducing preterm birth and neonatal complications via amniotic invasion. Poor oral health has also been linked to preterm labor through microbial translocation, with one study showing that women who underwent premature delivery had worse dental status and higher levels of *Fusobacterium nucleatum*, *Tannerella forsythia*, and *C. albicans* in their amniotic fluid, supporting an association between chronic oral infection and intrauterine microbial invasion [10].

Intra-amniotic *Candida* infections are correlated with preterm birth. In 773 women with preterm labor and intact membranes, 9.9% had positive amniotic cultures, with *Candida* in 6.5%. Among 625 women with premature rupture of membranes (PROM), 28% had positive cultures, with *Candida* in 2.2%, while no cases were found at term. Despite its common vaginal presence, *Candida* is an uncommon intra-amniotic pathogen, though it is linked to adverse obstetric outcomes [11]. Globally, fungal infections have risen steadily, particularly in immuno-compromised populations. A review spanning 1961–2024 showed increasing infection incidence and antifungal use, driving resistance and worsening outcomes. With an estimated 150 million infections and up to 3.8 million deaths annually, antifungal resistance is now a major public health concern, further aggravated by the limited arsenal of effective antifungals [12]. In this context, children and adolescents with hematologic malignancies are at high risk for invasive fungal diseases (IFDs). A 2013–2022 study in Heraklion reported a 7.8% IFD incidence, mainly of *Candida parapsilosis* and pulmonary aspergillosis, with 75% of these being breakthrough infections. The identified risk factors included prolonged neutropenia, extended ICU stays, fungal colonization, and the administration of multiple antimicrobials. Mortality remained high (16.7%), underscoring the need for better prevention and monitoring in pediatric hematology–oncology [13]. Emerging and uncommon *Candida* species are increasingly causing invasive infections in adults, complicating diagnosis and therapy. A review of 45 cases (2001–2023) identified *C. auris*, *C. haemulonii*, *C. fermentati*, and *C. kefyr* as the most frequent bloodstream isolates, with occasional recovery from pleural fluid and kidney tissue. Most of the considered reports were published in Asia and Europe, but species identification and susceptibility testing remain challenging due to the absence of standardized breakpoints [14]. A meta-analysis of 47 cohorts (> 91,000 patients with hematologic malignancies) found the highest incidence of invasive fungal diseases to be in allo-hematopoietic stem cell transplantation recipients, followed by those receiving chemotherapy or autologous transplantation. *Aspergillus* and *Candida* were the main pathogens, with *Candida* surpassing *Aspergillus* after 2010, though the shift was not statistically significant [15].

Despite progress in understanding intra-amniotic fungal infections, data on the antifungal resistance of *Candida* spp. in the amniotic compartment remain scarce. Resistant strains, limited drug penetration, and mechanisms such as *FKS* mutations and biofilm formation pose major challenges, underscoring the need to better define local susceptibility patterns to guide therapy in obstetric settings.

Little is known about the antifungal susceptibility of *Candida* spp. directly isolated from amniotic fluid; our study provides preliminary local data.

The aims of this study were as follows: (1) to evaluate the in vitro antifungal susceptibility of *Candida* species isolated from amniotic fluid using MIC-based testing; (2) to confirm the species-level identification of *C. albicans* via real-time PCR (RT-PCR); (3) to investigate potential associations between species distribution and temporal occurrence, antifungal resistance profiles, and relevant clinical variables such as patient age, gestational age, comorbidities, obstetric complications, and risk factors for preterm birth.

## 2. Materials and Methods

### 2.1. Study Design and Sample Collection

Between February and June 2025, a total of 40 *Candida* spp. strains were isolated from amniotic fluid samples collected for microbiological investigation at the “Cuza Vodă” Clinical Hospital of Obstetrics and Gynecology in Iași.

### 2.2. Inclusion and Exclusion Criteria

Patients were included if they were pregnant women aged > 16 years, regardless of pregnancy type, parity, fetal presentation, or associated comorbidities, who provided informed consent; exclusion criteria were samples with insufficient volume, missing essential clinical data, or repeated collections from the same patient.

### 2.3. Culture and Preliminary Identification

The specimens were cultured on Sabouraud dextrose agar (Oxoid, London, UK) and CHROMagar™ *Candida* (CHROMagar, Paris, France) to promote fungal growth and facilitate preliminary differentiation. Quality control was ensured by including the reference strain *Candida albicans* ATCC 10231 (Ref. 0443P, Lot 443-1662-42, exp. 30 November 2026), which was cultured in parallel under the same conditions.

### 2.4. Automated Identification and Antifungal Susceptibility Testing

Species-level identification and antifungal susceptibility testing were conducted using the VITEK^®^ 2 Compact system (bioMérieux, Paris, France), employing VITEK^®^ 2 YST cards for yeast identification and AST-YS08 cards for antifungal susceptibility testing. These automated tools provided standardized minimum inhibitory concentration (MIC) values for multiple antifungal agents, ensuring reliable and reproducible results. The antifungal agents included in the testing panel were fluconazole, voriconazole, micafungin, amphotericin B, caspofungin, and flucytosine. (bioMérieux, Paris, France)

Susceptibility interpretations were based on the latest available EUCAST clinical breakpoints (version 11.0, valid from 2 December 2024) to ensure standardized and clinically relevant categorization. For caspofungin, although the CLSI (M27M44S, 3rd ed., 2022) has established breakpoints, these were not applied as their inter-laboratory MIC variability means that EUCAST does not recognize them; thus, interpretation in a European context was based solely on EUCAST values. For flucytosine, neither EUCAST nor the CLSI currently provide breakpoints, as previous CLSI criteria were based on minimal clinical data and are no longer considered valid. Therefore, MIC values for both agents are reported without clinical categorization.

### 2.5. Molecular Confirmation by RT-PCR

In addition to phenotypic identification, RT-PCR was applied for molecular confirmation of species-level identification in a subset of 12 *C. albicans* isolates. For each isolate, the month of collection was recorded to allow assessment of temporal distribution.

#### 2.5.1. DNA Extraction

Genomic DNA was extracted using the genesig^®^ Easy DNA/RNA Extraction Kit (Primerdesign, London, UK), following the manufacturer’s magnetic-bead-based protocol. The process involved multiple purification steps: the initial lysis and binding stage (mixing 200 µL of sample with lysis buffer and internal extraction control), followed by sequential washes with Tubes 3 to 6, each involving the magnetic separation and removal of supernatant. After air drying, DNA was eluted in 200 µL of elution buffer (Tube 7) and recovered using a magnetic rack. The final elution volume used for downstream applications was 75 µL.

#### 2.5.2. PCR Assay

RT-PCR was performed using the genesig^®^ Advanced Kit for *Candida albicans* (Primerdesign Ltd., GENESIG, London, UK) which targets the *RPR1* gene and employs TaqMan^®^ probe technology. The kit comprises a fluorogenic 5′ nuclease assay that enables highly specific detection, the exact primer sequences being proprietary to the manufacturer but validated to have 100% homology with > 95% of *C. albicans* reference sequences in the NCBI database, as previously described by Innings et al. [16].

The PCR mix contained the lyophilized primers/probe, PrecisionPLUS 2X qPCR Master Mix, and 5 µL of DNA template, for a final volume of 20 µL per reaction. Thermal cycling was performed under the following conditions: enzyme activation at 95 °C for 2 min (1 cycle), denaturation at 95 °C for 10 s, and combined annealing/extension and data acquisition at 60 °C for 60 s (50 cycles). Fluorescent signals were recorded during the last step using the FAM channel.

A positive and negative control were included to validate amplification specificity. The positive control consisted of the *C. albicans* Positive Control Template supplied with the kit, while RNase/DNase-free water served as the negative control. For experimental validation, the positive control template was expected to amplify between Cq 16 and 23, and the negative should show no Ct. The RT-PCR assay was performed on an MX3005P Stratagene thermocycler.

### 2.6. Statistical Analysis

Statistical analysis was performed using IBM SPSS Statistics version 26.0 (IBM Corp., Armonk, NY, USA). Descriptive statistics were applied to both categorical and continuous variables. Frequencies and percentages were used to describe categorical data, such as *Candida* species distribution, antifungal susceptibility patterns, and patient characteristics. The Crosstabs function was used to explore distributions and cross-classifications between variables, and the Chi-square test was applied only to assess associations between *Candida* species and their antifungal susceptibility profiles (*p* < 0.05 was considered statistically significant), while the other variables were analyzed descriptively. For statistical analysis, only isolates with valid MIC values and interpretable categories according to EUCAST were included. Isolates without MIC values or with uninterpretable results were excluded from Chi-square/Fisher’s exact test analyses.

### 2.7. Ethical Considerations

Regarding informed consent, at all patients signed a consent form upon hospitalization authorizing the collection of biological samples for diagnostic purposes, including of amniotic fluid when clinically indicated. For this study, no additional sampling was performed; only data obtained from diagnostic procedures were used. Ethical approval was subsequently obtained from the Ethics Committee of the “Cuza Vodă” Clinical Hospital of Obstetrics and Gynecology, Iași (Approval No. 28/11/2024; opinion issued on 23 January 2025), and from the Ethics Committee of the “Grigore T. Popa” University of Medicine and Pharmacy, Iași (Approval No. 523/25 January 2025), for the anonymized scientific use of these data. All patient information remains strictly confidential.

## 3. Results

### 3.1. Species Distribution by Month, Age, Parity, Gravidity, and Associated Pathologies

Analysis of the monthly distribution showed that *C. albicans* predominated in every month, with the highest isolation in June (eight cases, 20% of all cases), while *P. kudriavzevii* was identified mainly in June (four cases, 10%). *N. glabratus* and *C. parapsilosis* were sporadically detected in March, April, and June (≤1 case each), and *C. dubliniensis* appeared only in March. Age group distribution analysis revealed that *C. albicans* was also the most frequent species across all intervals, most notably in patients aged 17–26 years (14 cases) and 27–31 years (5 cases), while *P. kudriavzevii* occurred mainly in 27–31-year-old patients (3 cases). *N. glabratus* was rarely identified in younger or older age groups, and *C. dubliniensis* and *C. parapsilosis* only appeared in isolated cases. Regarding parity, *C. albicans* was dominant in every category, especially in parities I (13 cases) and II (12 cases), with *P. kudriavzevii* more frequent in parities I (4 cases) and II (3 cases). *N. glabratus* was detected occasionally in parities I and III, while *C. dubliniensis* and *C. parapsilosis* were scarcely observed in parity II.

For gravidity, *C. albicans* was most often isolated in gravidities I (9 cases) and II (12 cases), with *P. kudriavzevii* following the same trend (3 and 4 cases, respectively). *N. glabratus* appeared sporadically in several gravidity categories, and *C. dubliniensis* and *C. parapsilosis* were only found in low numbers. Among associated maternal–fetal pathologies, *C. albicans* was detected most frequently in threatened preterm birth (9 cases), nuchal cord (5 cases), and other comorbidities such as morbid obesity, gestational diabetes, and autoimmune conditions. *P. kudriavzevii* was also present in threatened preterm birth (3 cases) and nuchal cord (2 cases), with occasional isolation in morbid obesity and placenta praevia. *N. glabratus* and *C. parapsilosis* contributed minimally across all pathological categories, and *C. dubliniensis* was only noted in isolated instances (Table 1). Species distribution was analyzed descriptively (frequencies and percentages), and no Chi-square test was performed.

### 3.2. Antifungal Susceptibility Profiles of Candida Species

Antifungal susceptibility testing: For fluconazole, there was a significant association between *Candida* species and susceptibility (*p* < 0.001). Most *C. albicans* isolates were susceptible, while *C. dubliniensis* showed full resistance. *C. parapsilosis* was fully susceptible, whereas *Pichia kudriavzevii* exhibited intrinsic resistance, with eight of eight isolates classified as resistant. For fluconazole, a significant association was observed between *Candida albicans* and non-*albicans Candida* (χ^2^ (1, *N* = 37) = 23.83, *p* < 0.001). Although 25% of the cells had expected counts <5, Fisher’s exact test confirmed the significance (*p* < 0.001). Additionally, non-*albicans Candida* isolates showed a markedly higher proportion of non-susceptibility compared to *C. albicans*. For fluconazole, twenty-four *Candida albicans* isolates were susceptible (MIC ≤ 0.5 µg/mL), one isolate was susceptible with an MIC of 2 µg/mL, and two isolates were resistant, with MICs of 8 µg/mL and ≥64 µg/mL; *Candida dubliniensis* was resistant (MIC = 16 µg/mL); and *C. parapsilosis* (*n* = 1) was susceptible, with an MIC of 1 µg/mL.

For voriconazole, susceptibility patterns differed significantly. None of the *C. albicans* isolates were fully susceptible, with 15 out of 27 requiring increased exposure (MIC ≤ 0.12 µg/mL). The single *C. dubliniensis* isolate also showed susceptibility at increased exposure (MIC ≤ 0.12 µg/mL), while *C. parapsilosis* was the only species with complete susceptibility (MIC ≤ 0.12 µg/mL).

For voriconazole, the Pearson Chi-square test indicated significance (χ^2^ (1, *N* = 17) = 7.97, *p* = 0.005); however, 75% of the cells had expected counts < 5, violating the test assumptions, and Fisher’s exact test, which is more appropriate for small sample sizes, did not confirm this association (*p* = 0.118). Therefore, no statistically significant difference was observed between *Candida albicans* and non-*albicans Candida* in terms of voriconazole susceptibility. For micafungin, significant variation was observed. The majority of *C. albicans* isolates were resistant, with only two being susceptible; *C. dubliniensis* was fully resistant; *N. glabratus* showed resistance in two out of three isolates (66.7%); and no susceptible isolates were observed among *P. kudriavzevii*. For micafungin, no significant association was observed between *Candida albicans* and non-*albicans Candida* (Pearson χ^2^ (1, *N* = 21) = 0.46, *p* = 0.496; Fisher’s exact test, *p* = 0.489), though due to the small number of isolates and the high proportion of expected counts < 5 (75%), these results should be interpreted with caution.

For micafungin, 2 *C. albicans* isolates were susceptible (MIC = 1 µg/mL), while 15 were resistant, including 13 isolates with MICs ≤ 0.06 µg/mL and 2 isolates with MICs of 0.12 µg/mL. The one *C. dubliniensis* isolate was resistant (MIC = 0.12 µg/mL), the single *C. parapsilosis* isolate was susceptible (MIC = 1 µg/mL), and two *N. glabratus* isolates were resistant (MICs ≤ 0.06 µg/mL).

For amphotericin B, susceptibility was significantly associated with species. Most *C. albicans* isolates were susceptible, with only two showing resistance, while *C. dubliniensis*, *C. parapsilosis*, and *N. glabratus* displayed full susceptibility. Resistance was observed in seven *P. kudriavzevii* isolates, representing the majority of this species. For amphotericin B, a significant association was observed between *Candida albicans* and non-*albicans Candida* (χ^2^ (1, *N* = 37) = 11.16, *p* = 0.001), and although 25% of the cells had expected counts <5, Fisher’s exact test confirmed the Chi-square result (*p* = 0.002). For amphotericin B, 19 *C. albicans* isolates were susceptible (MIC = 1 µg/mL), 4 were susceptible with an MIC = 0.5 µg/mL, and 2 were resistant, with MICs of 2 µg/mL and ≥16 µg/mL. The single *C. dubliniensis* isolate was susceptible (MIC = 0.5 µg/mL), as was the single *C. parapsilosis* isolate (MIC ≤ 0.25 µg/mL). *N. glabratus* showed susceptibility in all isolates, with two having an MIC ≤ 0.25 µg/mL and one with an MIC of 0.5 µg/mL. For *Pichia kudriavzevii*, seven isolates were resistant (MICs ≥ 16 µg/mL). Caspofungin susceptibility cannot be reliably interpreted using EUCAST criteria, as no official breakpoints have been established due to significant inter-laboratory MIC variability; interpretation is only acceptable when the isolate is susceptible to micafungin and anidulafungin. In this study, although five *Candida albicans* isolates showed low MICs (≤0.12 mg/L), most were resistant to micafungin, preventing interpretation. The *C. dubliniensis* isolate had a caspofungin MIC of 0.25 mg/L, classified as “IE” (insufficient evidence), meaning interpretation was not possible. The same applies to *P. kudriavzevii*, with MICs ≥ 8 mg/L and no established interpretive criteria (Table 2).

Although the CLSI has established breakpoints for caspofungin, antifungal susceptibility testing in Europe is interpreted according to EUCAST standards, which do not provide breakpoints for this drug. Therefore, caspofungin MIC values are reported without clinical categorization.

For flucytosine, although the analyzer provided MIC values, neither EUCAST nor the CLSI currently provide clinical breakpoints for this antifungal. In this study, five *C. albicans* isolates had MICs ≤ 1 mg/L, the single *C. dubliniensis* isolate had an MIC of 8 mg/L, and *Pichia kudriavzevii* showed an MIC of 16 mg/L. As no interpretive criteria are available, these MIC values are presented without clinical categorization.

### 3.3. Confirmation of C. albicans by RT-PCR

**Figure 1:**
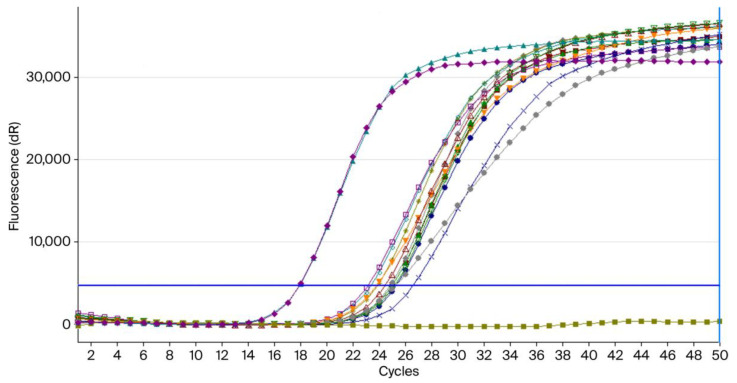
This RT-PCR amplification plot for *Candida albicans* shows a flat olive-green line for the negative control (no Ct), purple and green curves for duplicate positive controls (Ct = 18), and the rest are for clinical isolates (Ct 23–27).

The amplification plot shows the fluorescence curves obtained from real-time PCR targeting *C. albicans*. All 12 clinical samples exhibit exponential amplification, with Ct values ranging from approximately 23.1–26.6, indicating the presence of DNA/*C. albicans* (Figure 1). A complete explanation of this experiment can be found in the Supplementary Materials.

## 4. Discussion

### 4.1. Main Findings and Comparison with the Literature

This study provides, for the first time in our field, the antifungal susceptibility of *Candida* species isolated from amniotic fluid. *C. albicans* predominated (67.5%), followed by *P. kudriavzevii*, *N. glabratus*, *C. dubliniensis*, and *C. parapsilosis*. Most *C. albicans* isolates were susceptible to the tested antifungals. A notable case involved a 17-year-old primigravida at 35 weeks with PPROM and fetal anomalies, ending in antepartum fetal death. The *C. albicans* strain isolated showed micafungin resistance (MIC = 0.12 µg/mL), raising concerns about therapeutic efficacy. By contrast, Bové et al. [17] described the management of intra-amniotic *C. albicans* fluconazole, prolonging pregnancy and yielding a favorable neonatal outcome. The second notable case was a 20-year-old (G2P2) who presented at 31 weeks with imminent preterm birth and a structurally normal fetus. *C. albicans* was isolated, showing fluconazole resistance (MIC = 8 µg/mL), a rare but increasingly reported finding. In our case, preserved susceptibility enabled treatment with amphotericin B and voriconazole. By contrast, Nishino et al. [18] reported full-term *C. albicans* chorioamnionitis after untreated vaginal colonization, progressing to a maternal pelvic abscess despite a lack of susceptibility data, with *Candida* spp. confirmed in the placenta and cord. Our findings are similar to Korean data, with *C. albicans* predominant but rising incidences of non-*albicans Candida* species, particularly *N. glabratus* [19].

In our study, *N. glabratus* appeared in only three cases, indicating lower prevalence than in some reports. The clinical significance of fungal isolation in obstetric samples remains unclear. Berezowsky et al. [20] found low concordance between cultures and histological chorioamnionitis in preterm placentas, and although *N. glabratus* often shows resistance, our isolates were fully susceptible to amphotericin B. Patients frequently had risk factors such as twin pregnancy with diabetes, obesity, or placenta previa. Polat et al. included *Candida* spp. in their microbial invasion of the amniotic cavity (MIAC) investigations, but lacked species-level testing, while recent studies suggest that vaginal metabolomics could detect fungal MIAC noninvasively. In contrast, our *C. albicans* isolates remained fluconazole- and amphotericin B-susceptible, consistent with reports of high azole sensitivity in pregnancy (e.g., 93.2% miconazole susceptibility in vulvovaginal candidiasis) [21,22]. Our results agree with a those of a retrospective study of 663 pregnant women, where *C. albicans* was the most frequently isolated species, highly susceptible to azoles in the first trimester. By contrast, *C. nivariensis* was reported in an Indian woman with premature rupture of membranes (PROM), showing a high fluconazole MIC [23]. Initially misidentified as *N. glabratus* by VITEK^®^ 2, the species was later confirmed by MALDI-TOF and sequencing. The strain had a fluconazole MIC of 2 µg/mL, indicating reduced susceptibility, consistent with previous reports [23]. In pregnant women with vulvovaginal candidiasis (VVC), another study found a predominance of non-albicans strains, consistent with our rising rates of *N. glabratus*. In that cohort, *N. glabratus* comprised 54.4% of non-albicans isolates, followed by *C. dubliniensis* and *C. parapsilosis*, emphasizing the need for species-level identification to avoid treatment failure and recurrence [24]. In women with preterm PROM, a bedside test for matrix metalloproteinase-8 (MMP-8) in amniotic fluid strongly correlated with intra-amniotic inflammation severity and adverse outcomes. Higher MMP-8 values were linked to more infections, elevated inflammatory markers, and shorter time to delivery [25]. Recent studies show that α-bisabolol has antifungal activity against *C. albicans*, inhibiting adhesion, hyphal transition, biofilm formation, and ergosterol synthesis in a dose-dependent way, as well as inducing G1-phase arrest and gene expression changes, with strong binding to 14-α-demethylase and TUP1 [26].

Finally, our antifungal MIC distributions align with EUCAST recommendations [27], particularly for echinocandins, with isolates classified as “susceptible, increased exposure”, emphasizing the need for optimized dosing strategies. However, interpretation of caspofungin and flucytosine susceptibility remains limited by the absence of EUCAST breakpoints. Although the CLSI provides criteria for caspofungin, these are not applied in Europe, while for flucytosine, the CLSI no longer maintains valid breakpoints. This represents a limitation of our study, as MIC values for these agents cannot be reliably interpreted in our clinical context [28]. Detailed clinical information was available only for a subset of patients with comorbidities and increased risk of preterm birth, making them clinically relevant, while for the remaining cases, their medical charts reported no major risk factors or complications, restricting further knowledge of clinical context.

### 4.2. Clinical Implications: Management Challenges in Obstetrics, Need for Stewardship

The ESCMID Study Group for Antimicrobial Stewardship (ESGAP) was established in 1999 to promote responsible antibiotic use, leading to the formation of numerous clinical guidelines, including those on decolonization and brain abscess management [29,30]. In parallel, antifungal stewardship initiatives have been launched to optimize antifungal use across all care levels [31].

Maternal colonization with *C. albicans* was identified in 14.1% of pregnant women in Ethiopia, with vertical transmission occurring in 44.9% of cases, particularly among those with gestational diabetes or HIV [32]. Separately, intestinal dysbiosis with *C. albicans* and *Clostridioides difficile* overgrowth was more common in women with metabolic syndrome and very early preterm birth, suggesting a link between fungal imbalance and adverse outcomes [33].

In Italy, a five-year multicenter study found a decreasing prevalence of *C. albicans* and a rise in NAC species, with increased amphotericin B resistance in *C. parapsilosis* and continued high fluconazole resistance in *N. glabratus* [34].

These trends complicate treatment and reinforce the need for antifungal stewardship, especially as adherence to guidelines is often inconsistent in practice. Tools such as the paed-EQUAL *Candida* Score may aid clinicians in pediatric settings [35]. A Minnesota study (2019–2021) found high multidrug resistance rates in *N. glabratus*, highlighting the importance of local surveillance and within-patient variability [36]. New global guidelines from the ECMM, ISHAM, and ASM address emerging threats, including *C. auris* and taxonomy changes [37]. *C. albicans* was identified in a high-risk pregnancy with echogenic intra-amniotic material suggestive of infection. The absence of susceptibility testing limited therapeutic decision-making and underscores the need for antifungal stewardship [38]. Adjusting micafungin dosing to body surface area improves recovery in candidemia cases [39], while neonatal fungal infection prevention relies on bundles, fluconazole prophylaxis, catheter care, and early diagnosis [40].

At the Servicio de Micología in Madrid in 2010, Cuesta I. et al. compared EUCAST and CLSI fluconazole breakpoints for *Candida* spp., showing that reference methods produced similar MIC results. Using supervised classification algorithms to analyze the clinical data underlying each organization’s breakpoints, they found comparable outcomes, suggesting potential alignment between CLSI and EUCAST standards [41]. That same year, Pfaller M.A. et al. emphasized the need to harmonize these methodologies, noting that EUCAST cutoffs were species-specific, unlike CLSI’s broader application [42]. In Australia, van Hal S. J. et al. demonstrated a direct link between higher fluconazole MICs and mortality in *C. albicans* bloodstream infections, highlighting the clinical significance of revised EUCAST and CLSI breakpoints [43].

Twelve years later, both the CLSI and EUCAST established standardized broth microdilution methods and breakpoints (BPs) for in vitro antifungal susceptibility testing. Espinel-Ingroff A. reviewed the accuracy of commercial ECV-based methods such as E-test and SYO for detecting non-wild-type *Candida* species with resistance mutations. The review also addressed automated systems like Vitek-2^®^, the ATB^®^ fungus panel, and Fungitest^®^, which assess MICs for multiple antifungal agents, emphasizing that ECVs do not predict clinical response as BPs do and concluding that; therefore, BPs should be used whenever available for specific species/agent combinations [44]

### 4.3. Distinguishing True Infection from Contamination

Amniotic fluid is normally sterile, and fungal species detection requires cautious interpretation. Pregnancy involves a physiological shift toward immune tolerance, and several participants had conditions that may increase susceptibility to infection (such as autoimmune thrombocytopenia, type 1 diabetes mellitus, Rh incompatibility, or severe obstetrical complications including threatened preterm birth, cervico-isthmic incompetence, placenta praevia, and intrauterine growth restriction). Lass-Flörl C goes beyond existing guidelines to present practical insights that support clinicians and laboratorians in the diagnosis of invasive fungal infections, as well as outlining a context-driven approach to supplement current recommendations. For the diagnosis of yeast infections caused by *Candida* species, Lass-Flörl discusses relevant clinical specimens (such as blood, peritoneal fluid, and CSF) and several diagnostic methods, including microscopy (rapid detection with variable sensitivity around 50–60% and high specificity, above 90%), culture (blood culture, with a sensitivity of approximately 50–70% and near-100% specificity), PCR methods (multiplex *Candida* real-time PCR for direct blood testing, with 84% sensitivity and 33% specificity), and biomarkers (β-D-glucan, with sensitivity near 85% and specificity around 76%).

The article notes that combining multiple diagnostic tools, including antigen tests, cultures, imaging, and evaluation of host risk factors, can increase diagnostic accuracy for detecting truly invasive disease. This stepwise, integrative diagnostic methodology that incorporates various approaches and considers host factors is consistent with current guideline recommendations [45].

### 4.4. Study Limitations and Future Directions

This study has several limitations. First, as a prospective pilot study, the number of samples included was relatively small, particularly for non-*albicans Candida* species, which reduces the statistical power and generalizability of our findings. As the Fisher exact and Chi-square tests were applied despite these small cell counts, we mention the limitations of the power and validity of our statistical analysis. Second, molecular confirmation was only performed for *C. albicans* isolates due to the availability of an RT-PCR kit specific to this species. We acknowledge that extending molecular identification to all isolates, including non-*albicans Candida*, would strengthen the reliability of the results. Third, antifungal susceptibility testing was conducted and interpreted according to EUCAST standards, which represent the routine practice in Romania; however, confirmation with reference methods such as CLSI broth microdilution or the E-test was not performed. Furthermore, distinguishing true intra-amniotic infection from colonization remains inherently challenging, and this study highlights the need for integrated clinical and microbiological approaches to address this issue in future research. Finally, clinical correlations with pregnancy or neonatal outcomes were not systematically assessed in this pilot phase. Despite these limitations, this study provides novel preliminary data on the antifungal susceptibility of *Candida* spp. isolated from amniotic fluid, highlighting the need for larger, multicenter studies to validate these findings and further elucidate the impact of intra-amniotic *Candida* infections on maternal and neonatal outcomes.

## 5. Conclusions

To our knowledge, this study is the first to highlight the microbiological and clinical heterogeneity of intra-amniotic *Candida* infections in Northeast Romania. *C. albicans* was identified as the main species isolated from amniotic fluid, and the detection of non-*albicans* strains, including *P. kudriavzevii*, *N. glabratus*, *C. parapsilosis,* and *C. dubliniensis*, supports the importance of accurate species-level identification. While most isolates were either susceptible to or in the increased-exposure group for voriconazole, there were still two cases that showed micafungin resistance. Amphotericin B sensitivity was detected in most isolates, supporting its continued use in antifungal stewardship.

## Figures and Tables

**Table 1:** Distribution of cases by demographic and clinical characteristics and associated maternal–fetal pathologies according to *Candida* species (*n*, %).

Characteristic	Total *n* (%)	*C. albicans* *n* (%)	*N. glabratus* *n* (%)	*C. parapsilosis* *n* (%)	*C. dubliniensis* *n* (%)	*P. kudriavzevii n* (%)
Distribution by month						
February	8 (20.0%)	6 (15.0%)	2 (5.0%)	0 (0.0%)	0 (0.0%)	0 (0.0%)
March	10 (25.0%)	7 (17.5%)	0 (0.0%)	0 (0.0%)	1 (2.5%)	2 (5.0%)
April	3 (7.5%)	2 (5.0%)	0 (0.0%)	0 (0.0%)	0 (0.0%)	1 (2.5%)
May	5 (12.5%)	4 (10.0%)	0 (0.0%)	0 (0.0%)	0 (0.0%)	1 (2.5%)
June	14 (35.0%)	8 (20.0%)	1 (2.5%)	1 (2.5%)	0 (0.0%)	4 (10.0%)
Distribution by age group (5-year intervals)						
17–21 years	7 (17.5%)	7 (17.5%)	0 (0.0%)	0 (0.0%)	0 (0.0%)	0 (0.0%)
22–26 years	9 (22.5%)	7 (17.5%)	0 (0.0%)	0 (0.0%)	0 (0.0%)	2 (5.0%)
27–31 years	10 (25.0%)	5 (12.5%)	0 (0.0%)	0 (0.0%)	2 (5.0%)	3 (7.5%)
32–36 years	6 (15.0%)	3 (7.5%)	0 (0.0%)	0 (0.0%)	1 (2.5%)	2 (5.0%)
37–41 years	8 (20.0%)	5 (12.5%)	1 (2.5%)	1 (2.5%)	0 (0.0%)	1 (2.5%)
Parity						
I	20 (50.0%)	13 (32.5%)	2 (5.0%)	0 (0.0%)	1 (2.5%)	4 (10.0%)
II	16 (40.0%)	12 (30.0%)	0 (0.0%)	1 (2.5%)	0 (0.0%)	3 (7.5%)
III	2 (5.0%)	1 (2.5%)	1 (2.5%)	0 (0.0%)	0 (0.0%)	0 (0.0%)
IV	1 (2.5%)	0 (0.0%)	0 (0.0%)	0 (0.0%)	0 (0.0%)	1 (2.5%)
VII	1 (2.5%)	1 (2.5%)	0 (0.0%)	0 (0.0%)	0 (0.0%)	0 (0.0%)
Gravidity						
I	14 (35.0%)	9 (22.5%)	1 (2.5%)	0 (0.0%)	1 (2.5%)	3 (7.5%)
II	18 (45.0%)	12 (30.0%)	1 (2.5%)	1 (2.5%)	0 (0.0%)	4 (10.0%)
III	4 (10.0%)	4 (10.0%)	0 (0.0%)	0 (0.0%)	0 (0.0%)	0 (0.0%)
IV	2 (5.0%)	1 (2.5%)	1 (2.5%)	0 (0.0%)	0 (0.0%)	0 (0.0%)
V	1 (2.5%)	0 (0.0%)	0 (0.0%)	0 (0.0%)	0 (0.0%)	1 (2.5%)
VII	1 (2.5%)	1 (2.5%)	0 (0.0%)	0 (0.0%)	0 (0.0%)	0 (0.0%)
Associated maternal–fetal pathologies						
Threatened preterm birth	14 (35.0%)	9 (22.5%)	1 (2.5%)	1 (2.5%)	0 (0.0%)	3 (7.5%)
Nuchal cord	8 (20.0%)	5 (12.5%)	1 (2.5%)	0 (0.0%)	0 (0.0%)	2 (5.0%)
Morbid obesity	3 (7.5%)	1 (2.5%)	0 (0.0%)	0 (0.0%)	0 (0.0%)	2 (5.0%)
Gestational diabetes	3 (7.5%)	2 (5.0%)	0 (0.0%)	0 (0.0%)	0 (0.0%)	1 (2.5%)
Rh incompatibility	2 (5.0%)	2 (5.0%)	0 (0.0%)	0 (0.0%)	0 (0.0%)	0 (0.0%)
Type 2 diabetes mellitus	2 (5.0%)	2 (5.0%)	0 (0.0%)	0 (0.0%)	0 (0.0%)	0 (0.0%)
Threatened miscarriage	2 (5.0%)	2 (5.0%)	0 (0.0%)	0 (0.0%)	0 (0.0%)	0 (0.0%)
Marginal placenta praevia	2 (5.0%)	1 (2.5%)	0 (0.0%)	0 (0.0%)	0 (0.0%)	1 (2.5%)
Cervico-isthmic incompetence	2 (5.0%)	0 (0.0%)	1 (2.5%)	1 (2.5%)	0 (0.0%)	0 (0.0%)
PIH *	2 (5.0%)	1 (2.5%)	0 (0.0%)	0 (0.0%)	0 (0.0%)	1 (2.5%)
Subchorionic hematoma	1 (2.5%)	1 (2.5%)	0 (0.0%)	0 (0.0%)	0 (0.0%)	0 (0.0%)
Vaginitis	1 (2.5%)	1 (2.5%)	0 (0.0%)	0 (0.0%)	0 (0.0%)	0 (0.0%)
Autoimmune thrombocytopenia	1 (2.5%)	1 (2.5%)	0 (0.0%)	0 (0.0%)	0 (0.0%)	0 (0.0%)
Genital bleeding	1 (2.5%)	1 (2.5%)	0 (0.0%)	0 (0.0%)	0 (0.0%)	0 (0.0%)
Fetal ultrasound anomalies	1 (2.5%)	1 (2.5%)	0 (0.0%)	0 (0.0%)	0 (0.0%)	0 (0.0%)
Large for gestational age	1 (2.5%)	0 (0.0%)	0 (0.0%)	0 (0.0%)	0 (0.0%)	1 (2.5%)
IUGR **	1 (2.5%)	1 (2.5%)	0 (0.0%)	0 (0.0%)	0 (0.0%)	0 (0.0%)
Reversed Doppler indices	1 (2.5%)	1 (2.5%)	0 (0.0%)	0 (0.0%)	0 (0.0%)	0 (0.0%)
Type 1 IDDM ***	1 (2.5%)	0 (0.0%)	0 (0.0%)	0 (0.0%)	0 (0.0%)	1 (2.5%)
Autoimmune thyroiditis	1 (2.5%)	1 (2.5%)	0 (0.0%)	0 (0.0%)	0 (0.0%)	0 (0.0%)

Notes: * Pregnancy-induced hypertension; ** intrauterine growth restriction; *** type 1 insulin-dependent diabetes mellitus.

**Table 2:** Antifungal susceptibility profiles of *Candida* species to fluconazole, voriconazole, micafungin, and amphotericin B (*n*, %, within species; total row calculated from *N* = 40).

Species/Antifungal	Fluconazole		Voriconazole		Micafungin		Amphotericin B	
	Susceptible *n* (%)	Resistant *n* (%)	Susceptible *n* (%)	Susceptible, IP * *n* (%)	Susceptible *n* (%)	Resistant *n* (%)	Susceptible *n* (%)	Resistant *n* (%)
*Candida albicans*	≤2 mg/L	>4 mg/L	≤0.06 mg/L	>0.06–≤0.25 mg/L	≤0.03 mg/L	>0.03 mg/L	≤1 mg/L	>1 mg/L
	25/27 (92.6%)	2/27 (7.4%)	0 (0.0%)	15/27 (55.6%)	2/27 (7.4%)	15/27 (55.6%)	23/27 (85.3%)	2/27 (7.4%)
*Candida dubliniensis*	≤2 mg/L	>4 mg/L	≤0.06 mg/L	>0.06–≤0.25 mg/L	≤0.06 mg/L	>0.06 mg/L	≤1 mg/L	>1 mg/L
	0/1 (0.0%)	1/1 (100%)	0/1 (0.0%)	1/1 (100%)	0/1 (0.0%)	1/1 (100%)	1/1 (100%)	0/1 (0.0%)
*Candida parapsilosis*	≤2 mg/L	>4 mg/L	≤0.125 mg/L	>0.125–≤0.25 mg/L	≤4 mg/L	>4 mg/L	≤1 mg/L	>1 mg/L
	1/1 (100%)	0/1 (0.0%)	1/1 (100%)	0/1(0.0%)	1/1 (100%)	0/1 (0.0%)	1/1 (100%)	0/1 (0.0%)
*Nakaseomyces glabratus*	≤0.001 mg/L	>16 mg/L	-	-	≤0.03 mg/L	>0.03 mg/L	≤1 mg/L	>1 mg/L
	0/3 (0.0%)	0/3 (0.0%)	0/3 (0.0%)	0/3 (0.0%)	0/3 (0.0%)	2/3 (66.7%)	3/3 (100%)	0/3 (0.0%)
*Pichia kudriavzevii*	-	-	-	-	-	-	≤1 mg/L	>1 mg/L
	0/8 (0.0%)	8/8 (100%)	0/8 (0.0%)	0/8 (0.0%)	0/8 (0.0%)	0/8 (0.0%)	0/8 (0.0%)	7/8 (87.5%)
Total	26 (65.0%)	11 (27.5%)	1 (2.5%)	16 (40.0%)	3 (7.5%)	18 (45.0%)	28 (70.0%)	9 (22.5%)

Notes: Percentages in the ‘Total’ row are calculated relative to all isolates (*N* = 40), while species-specific rows are presented per species. For species with few isolates, values are shown as x/*n* (%). * Susceptible, increased exposure.

## Data Availability

The original contributions presented in this study are included in the article.

## Supplementary Materials

The following supporting information can be downloaded at https://www.mdpi.com/article/10.3390/pathogens14100972/s1: Table S1: cycle threshold (Ct) values obtained for clinical samples and controls by RT-PCR.
