# Supplementary material for: Candida Susceptibility to Antifungals in Amniotic Fluid: A Preliminary Study
Source: Pathogens. 2025 Sep 25;14(10):972. doi: 10.3390/pathogens14100972 (PMC12567086; doi:10.3390/pathogens14100972)
Supplement: Supplementary file 1 [file pathogens-14-00972-s001.zip › pathogens-3884041-supplementary.pdf]

Supplementary material

Plate layout and Ct values for *C. albicans* detection using real-time PCR: all 12 samples (wells C4–C9 and E4–E9) amplified with Ct values ranging from 23.1 to 26.6, indicating positive detection. The experiment was validated by adequate Ct control values (negative and positive). The negative control (olive green) remained below the threshold, while positive controls (purple and green) amplified early. Clinical samples crossed the threshold, confirming *Candida albicans* DNA detection.

The table summarizes the Ct values for the 12 clinical isolates tested, along with the no-template control (NTC) and the positive controls. Clinical samples amplified with Ct values ranging from 23.11 to 26.6, while positive controls amplified early (Ct = 17.91). The negative control showed no amplification (Table 1).

Table 1. Cycle threshold (Ct) values obtained for clinical samples and controls by RT-PCR

Replicate	Dye	Well Type	Ct	Replicate	Dye	Well Type	Ct
1	FAM	Unknown	25.3	9	FAM	Unknown	26.6
2	FAM	Unknown	24.99	10	FAM	Unknown	24.48
3	FAM	Unknown	24.98	11	FAM	Unknown	25.19
4	FAM	Unknown	24.86	12	FAM	Unknown	25.24
5	FAM	Unknown	23.89	H1	FAM	NTC (negative template control)	No Ct
6	FAM	Unknown	23.37				
7	FAM	Unknown	23.11	H11	FAM	FAM Positive Control	17.91
8	FAM	Unknown	23.81	H12	FAM	FAM Positive Control	17.91
